# Phase Diagram, Glassy Dynamics and Crystallization Kinetics of the Biobased Polyester Poly(ethylene 2,5-furanoate) (PEF)

**DOI:** 10.1021/acs.macromol.4c01962

**Published:** 2024-10-04

**Authors:** Ioannis Tzourtzouklis, Panagiotis Kardasis, George Z. Papageorgiou, George Floudas

**Affiliations:** † Department οf Physics, University οf Ioannina, Ioannina 45110, Greece; ‡ Department of Chemistry, University of Ioannina, Ioannina 45110, Greece; § University Research Center of Ioannina (URCI)-Institute of Materials Science and Computing, Ioannina 45110, Greece; ∥ Max Planck Institute for Polymer Research, Mainz 55128, Germany

## Abstract

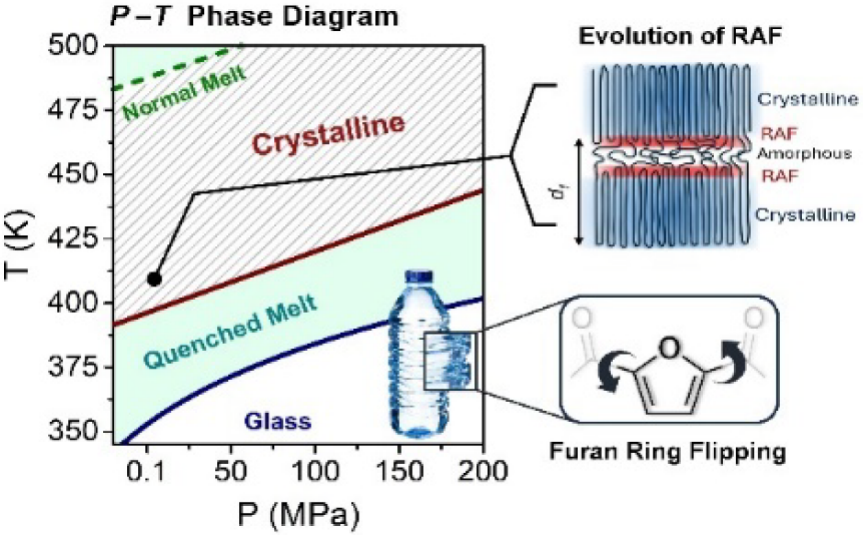

We report the pressure–temperature (*P*–*T*) phase diagram, the origin of the subglass dynamics, and the crystallization kinetics of the biobased polyester poly(ethylene 2,5-furanoate) (PEF), through dielectric spectroscopy (DS) measurements performed as a function of temperature and pressure. The phase diagram comprises four different “phases”; glass, quenched melt, crystalline, and normal melt. The cold crystallization temperature, *T*_cc_, increases linearly with pressure (according to the Clausius–Clapeyron equation) as *dT*_cc_/*dP*_|_*_P_*_→0_ ∼ 240 K·GPa^–1^ and is accompanied by a small change in specific volume (Δ*V* = 0.028 cm^3^/g). This contrasts with the stronger dependence of the glass temperature, *T*_*g*_, with a pressure coefficient, *dT*_*g*_/*dP*_|_*_P_*_→0_, of 383 K·GPa^–1^, typical of rigid polymers. With the application of pressure, we address the molecular origin of the subglass β-process through the apparent activation volume, a quantity accessible only by pressure experiments. Moreover, increasing pressure densifies the segmental process but blocks the β-process, with possible implications in the gas-barrier properties. The crystallization kinetics from the quenched melt to the cold-crystallized state was explored by thermodynamics (differential scanning calorimetry, DSC), dynamics (DS), and structure (via simultaneous X-ray scattering at small (SAXS) and wide (WAXS) angles) following different routes within the phase diagram. Interestingly, all probes followed the same sigmoidal kinetics (of the Avrami type) with comparable time scales. Inspection of the evolution of the dielectric strength for the different dynamic processes during isothermal crystallization (at *T*_c_ = 402 K; *P* = 0.1 MPa) revealed the absence of the *restricted amorphous fraction* (RAF) at the early stages of crystallization. This observation is in line with the proposed *mesomorphic phase*—an intermediate phase formed during crystallization in the absence of chain folding, as suggested by G. Strobl. Subsequent growth of the RAF followed the same Avrami kinetics as identified by the thermodynamic and structural probes. Shallow quenches within the *P*–*T* phase diagram identified experimental routes for keeping PEF in the metastable quenched amorphous state for long times.

## 1 Introduction

Poly(*n*-methylene 2,5-furanoates) is a family of biobased polymers with exceptional gas-barrier and mechanical properties.^1−7^ Poly(ethylene 2,5-furanoate) (PEF) in particular constitutes a 100% biobased and recyclable polyester, composed of the 2,5-furandicarboxylic acid and ethylene glycol. PEF has gained increasing interest by competing with petroleum-based PET in the packaging of liquids. This reflects on the enhanced thermal (higher *T*_*g*_, lower *T*_*m*_),^8−10^ mechanical (higher G*)^5^ and gas-barrier properties (much lower *CO*_2_ permeability).^10^ One peculiarity of the amorphous state of several poly(*n*-methylene 2,5-furanoates), including PEF, is the presence of compact helical structures that are stabilized by π–π interactions of the furan rings.^11,12^ The helical motifs influence the dynamics of amorphous segments, including the average relaxation time, the distribution of relaxation times, the dielectric strength, and fragility.^11,13^ In addition, given the high glass temperature of PEF (*T*_g_ ∼ 353 K), subglass processes (such as the β-process) are likely to be linked with the superior gas-barrier properties.^13^ These observation initiated several thermodynamic,^11,13−15^ dynamic^11,13,16−19^ and structural^20,21^ investigations of poly(*n*-methylene 2,5-furanoates), including PEF, as well as a series of investigations in furan-based polyester blends based on PEF.^22 −24^ In the latter case, as a general rule, dynamically homogeneous mixtures emerged (e.g., having a single liquid-to-glass temperature, *T*_g_) when the poly(*n*-methylene 2,5-furanoate) backbones differ by a single methylene unit.^23^ The dynamic studies of PEF in particular, have shown the existence of a restricted amorphous fraction (RAF),^13,14,25^ where a fraction of amorphous segments located at the interface of amorphous and crystalline domains forming a new “interphase” that comprises a large fraction of the semicrystalline state.

Pressure, in addition to temperature, plays an important role in the crystallization and gas-barrier properties of polymer used in bottle formation and recycling. Carbonated beverages in polymer packaging exhibit pressures of up to 0.5 MPa (5 bar) at ambient temperature.^26^ Processing of PET during the extrusion process requires pressures of up to 14 MPa.^27^ Chemical recycling of PET also involves high pressures (typically 2–4 MPa) and high temperatures. In addition, high temperatures and high pressures (∼15 MPa) are required in order to improve yield in PET hydrolysis, methanolysis, and glycolysis processes.^26^

Motivated by these effects, we employ for the first time pressure, in addition to temperature, dielectric spectroscopy measurements, to address (a) the pressure dependence of the segmental dynamics and (b) the origin of the secondary β-process associated with the exceptional gas-barrier properties of PEF. With the application of pressure, we further construct (c) the *P*–*T* phase diagram pertinent to processing. In the last part, we explore (d) the crystallization kinetics by following different paths in the phase diagram involving both temperature and pressure jumps. For one of these paths, involving a temperature jump from the quenched-amorphous state to the crystalline state, we compare the crystallization kinetics by means of structure (X-rays) at the relevant length scales, thermodynamics (with differential scanning calorimetry, DSC), and dynamics (by dielectric spectroscopy, DS). The comparison brings some new insights on how the evolution of the crystallization process affects the segmental dynamics within the different—mobile and less mobile—fractions. In particular, we show that the RAF is absent at the early stages of the crystallization process. It forms later during the course of crystallization and follows typical (Avrami-type) crystallization kinetics. We discuss this finding with respect to the proposed intermediate mesomorphic phase—in the absence of chain folding—formed at the initial stages of the crystallization (according to Strobl).^28^

## 2 Experimental Section

### 2.1 Synthesis

The synthesis of PEF was reported earlier.^29^ Briefly, 2,5-furan dicarboxylic acid (purum 97%) was purchased from Aldrich Co. Ethylene glycol and a tetrabutyl titanate (TBT) catalyst of analytical grade were also purchased from Aldrich Co. First 2,5-dimethylfuran-dicarboxylate (DMFD) was prepared by applying known procedures. The yield of the process was typically 83%. Subsequently, PEF was prepared by the two-stage melt polycondensation method (esterification and polycondensation), as described earlier. The intrinsic viscosity [η] of the sample is 0.54 dL/g and its molecular weight is 12 700 g/mol.

### 2.2 X-ray Scattering

**Figure 1:**
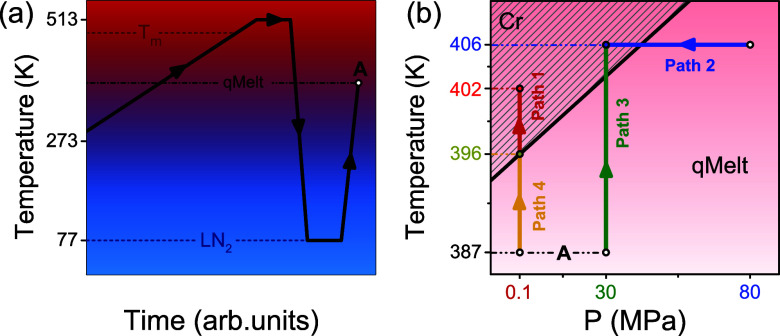
(a) Thermal protocol used in the preparation of completely amorphous PEF *(quenched amorphous)*. The sample was initially heated to 513 K (melt state) at ambient pressure and subsequently quenched below its glass temperature. DS measurements were obtained *o*n heating. (b) Different temperature–pressure paths for an initially quenched amorphous state (qMelt) (following the previous protocol) employed for the crystallization kinetics.

XRD measurements were made using CuKα radiation (λ = 1.54184 nm) with a Bruker D8 ADVANCE 2θ diffractometer, equipped with the detector LYNXEYE XE-T. The sample was prepared with the thermal protocol of Figure 1. Subsequently, the quenched sample was crystallized under isothermal conditions at *T*_*c*_ = 402 K and measured in the form of a thin film (300 μm) at room temperature in the 2θ (deg) range of 2°–60° in steps of 0.01° for 3600 s.

### 2.3 Small/Wide Angle X-ray Scattering (SAXS/WAXS)

The lamellar morphology of the crystalline PEF was detected through SAXS measurements, which were performed with the N8 Horizon vertical setup (Bruker), using a 50W CuKα radiation (IμS microfocus source with integrated MONTEL optics), in the *q* range of 0.1–3.7 nm^–1^. The scattered intensity was recorded on a VÅNTEC-500 2D detector at a distance of 660 mm away from the sample. Simultaneously with SAXS, WAXS measurements were performed with the VÅNTEC-1 detector in the *q* range of 13.8–19.2 nm^–1^. The sample was measured in the form of film (300 μm) and prepared again using the protocol of Figure 1. The quenched sample was then measured for 900 s under isothermal conditions for a total time of *t* = 10800 s (12 measurements).

### 2.4 Differential Scanning Calorimetry (DSC)

Isothermal crystallization kinetics of PEF were also investigated by differential scanning calorimetry through the QA 2000 (TA Instruments). The instrument was calibrated for the baseline using a sapphire standard, for the enthalpy and temperature using indium as a standard, and for the heat capacity using sapphire as a standard. The sample was heated to 513 K and then cooled to 273 K with an effective rate of 50 K·min^–1^ and eventually heated, with the same rate, up to 402 K. The whole process was implemented inside the measuring chamber. Isothermal crystallization was indicated as an increase and a subsequent decrease in the heat flow over time. Following the isothermal measurement, at 402 K for *t* = 10800 s, the sample was heated with a rate of 10 K·min^–1^ up to 523 K.

### 2.5 Dielectric Spectroscopy (DS)

Dielectric spectroscopy (DS) measurements were performed with a Novocontrol BDS system with a frequency response analyzer (Solatron Schlumberger FRA 1260) for frequencies in the range from 1 × 10^–2^ to 1 × 10^7^ Hz. Both “isobaric” measurements as a function of temperature and “isothermal” measurements as a function of pressure were made. The “isobaric” measurements were made at pressures of 0.1, 30, 50, and 80 MPa. The sample was heated up to 513 K (melt state) and subsequently quenched by immersion in liquid nitrogen to prevent crystallization (Figure 1b).

In all cases, the quenched amorphous PEF (following the preparation protocol shown in Figure 1a) was slowly heated, and the dielectric function, ε*(ω), was recorded. The dielectric cell for the *T*-dependent dielectric measurements consisted of PEF films with a thickness of ∼50 μm. Measurements under hydrostatic pressure were carried out in a Novocontrol pressure cell. The pressure setup consisted of an *T*-controlled cell, a hydraulic closing press with an air pump, and an air pump for hydrostatic test pressure. For the *P*-dependent measurements, PEF samples were pressed at the molten state between 20 mm diameter electrodes, and Teflon spacers were used to maintain a thickness of 50 μm. Subsequently, the capacitor was wrapped with Teflon tape and placed inside a Teflon ring in order to prevent the flow of silicone oil (DOW CORNING 550 Fluid) into the sample. The silicone oil is the liquid that uniformly transmits the pressure to the capacitor. The isothermal measurements of relaxation times were performed with temperature stability better than 0.1 K and pressure stability better than 2 MPa. In addition, the crystallization kinetics were studied via “isothermal” and “isobaric” *P*- and *T*- jumps, respectively, within the established *P*–*T* phase diagram. In each case, the quenched amorphous sample was placed inside the pressure cell and the crystallization kinetics were followed by specific *T*-jumps and *P*-jumps within preselected paths (Figure 1b).

Both in “isothermal” and “isobaric” measurements, the complex dielectric function, ε* = ε′– iε″, where ε′ is the real and ε″ is the imaginary part, was obtained as a function of frequency, ω, temperature, *T*, and pressure, *P*, i.e., ε*(*T*, *P*, ω).^30−33^ The analysis of the relaxation dynamics was made by using a summation of the empirical equation of Havriliak and Negami (HN):
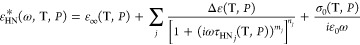
1where ε_∞_(*T*,*P*) is the high frequency permittivity, ε_0_(*T*,*P*) is the permittivity of free space, *τ*_*HN*_ (*T,P*) refers to the characteristic relaxation time of this model,  is the relaxation strength of the process under investigation, σ_0_(*T*,*P*) introduces the DC conductivity, *m* and *n* describe respectively the symmetrical and asymmetrical broadening of the relaxation times distribution, and ω (*= 2π f =* 1*/τ*) is the angular frequency of the external electric field. From the *τ*_*HN*_, the relaxation times at maximum loss, *τ*_max_, were obtained analytically from the HN equation as follows:
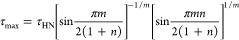
2

## 3 Results and Discussion

### 3.1 Segmental Dynamics

**Figure 2:**
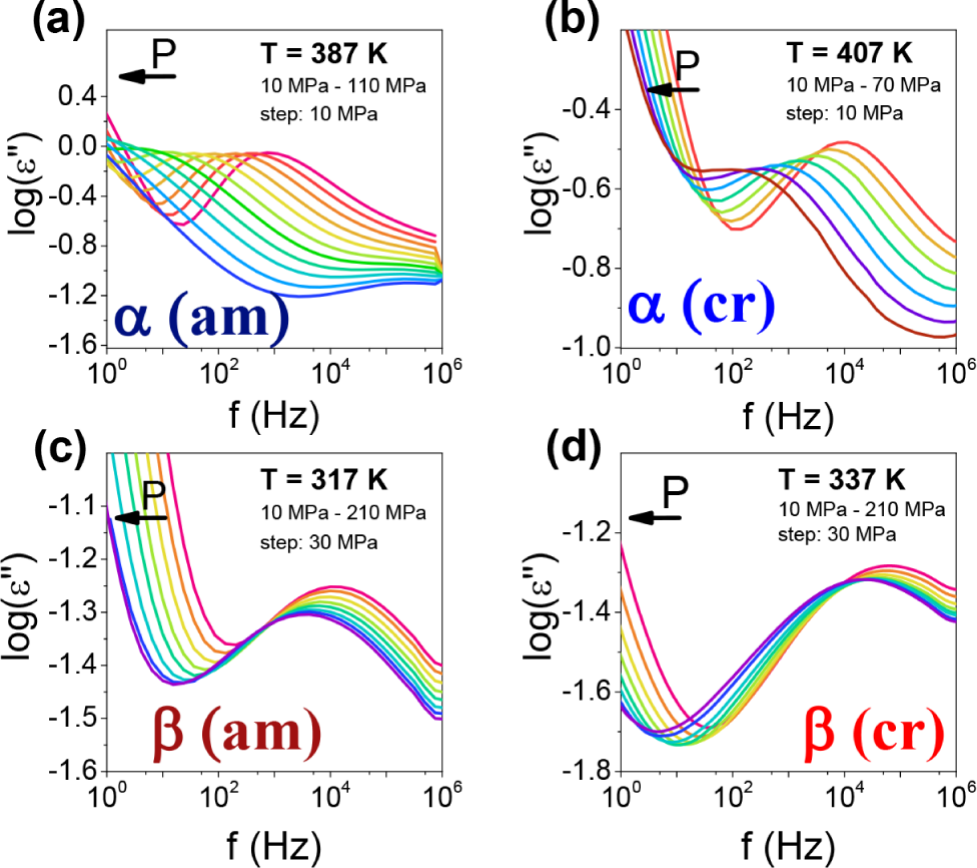
Dielectric loss curves for the segmental (α-process) and the local (β-process) in both the quenched amorphous (a,c) and semicrystalline (b,d) states at some selected temperatures: (a) At *T* = 387 K the dielectric loss curves correspond to the α-process in the quenched amorphous sample shown for pressures in the range from 10 to 110 MPa in 10 MPa steps. (b) At *T* = 407 K the curves correspond to the same α-process but in the semicrystalline state, shown for pressures in the range from 10 to 70 MPa, in 10 MPa steps. (c) The β-process in the quenched amorphous state is shown at *T* = 317 K, for pressures in the range from 10 to 210 MPa in 30 MPa steps. (d) The β-process in the semicrystalline state is shown at *T* = 337 K and within the same pressure range.

Dielectric measurements of the molecular dynamics of PEF reveal two dielectrically active processes for the quenched sample; a segmental (α-process), associated with the relaxation of the amorphous segments (α(am)) and a weaker process in the glassy state (β-process). On heating the quenched melt sample undergoes cold crystallization, and the α(am), is replaced by the segmental process in the semicrystalline state, indicated as α(cr). The latter reflects the dynamics of the amorphous segments that are restricted by the crystalline domains. A slower process reflecting the segmental dynamics within the restricted amorphous fraction (RAF) can be detected as a separate process following specific temperature (and pressure) protocols (see below with respect to the crystallization kinetics). Some representative dielectric loss curves as a function of pressure, are shown in Figure 2a,b for the segmental process in the amorphous α(am) and in the semicrystalline states, α(cr), respectively. Pressure slows down the dynamics of both processes; however, the effect is very prominent for the α-process both in the quenched amorphous and semicrystalline PEF.

**Figure 3:**
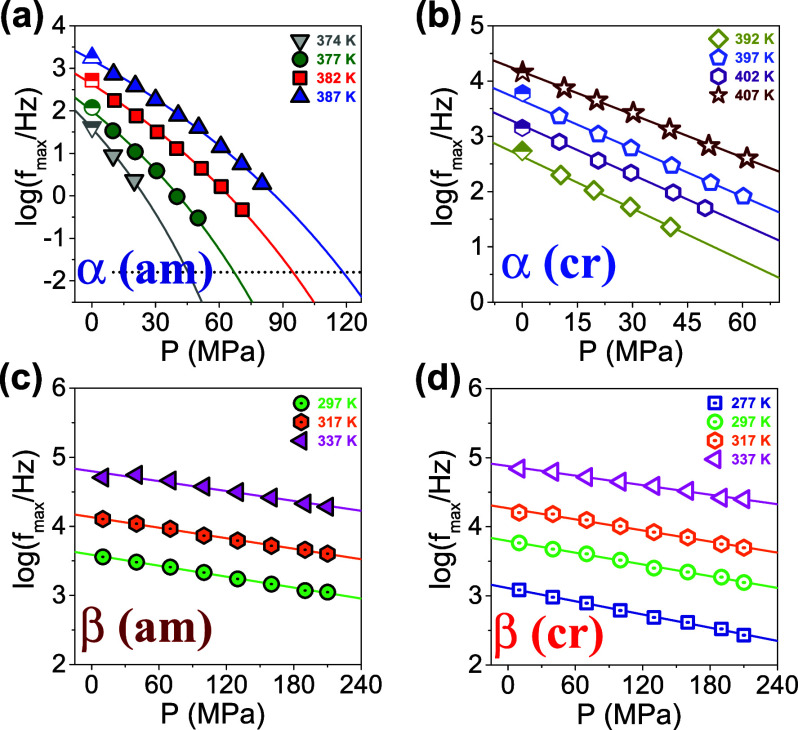
Characteristic frequencies at maximum loss plotted as a function of pressure for different temperatures for (a) the α-process in the quenched amorphous state α(am), (b) α-process in the semicrystalline state α(cr), (c) β-process in the amorphous state β(am), and (d) β-process in the semicrystalline state, β(cr). Different symbols and colors correspond to different temperatures as indicated. Filled symbols depict characteristic relaxation frequencies in the amorphous state, while open ones depict characteristic relaxation frequencies in the semicrystalline state. Half-filled symbols in (a,b) represent characteristic relaxation frequencies obtained at ambient pressure.

The pressure dependence of the characteristic frequencies at maximum loss corresponding to the segmental and β-processes are very distinct for the melt quenched and crystallizable PEF. This is shown in Figure 3 for the quenched amorphous PEF. The relaxation frequencies of the α-process follow the pressure counterpart of the VFT equation^31^ (Table S1) as
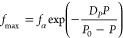
3where *f*_*α*_ is the segmental relaxation frequency at atmospheric pressure at a given temperature, *D*_*P*_ is a dimensionless parameter, and *P*_0_ is the pressure that corresponds to the “ideal” glass. The segmental dynamics in the semicrystalline state, α(cr), is depicted in Figure 3b, exhibiting a weaker *P*-dependence. This reflects the restrictions induced by the effect of crystallization in the segmental dynamics (as will be discussed later).

Pressure-dependent measurements can be used to extract the apparent activation volume, Δ*V*^*#*^, corresponding to the underlying processes as^31^
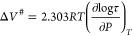
4

**Figure 4:**
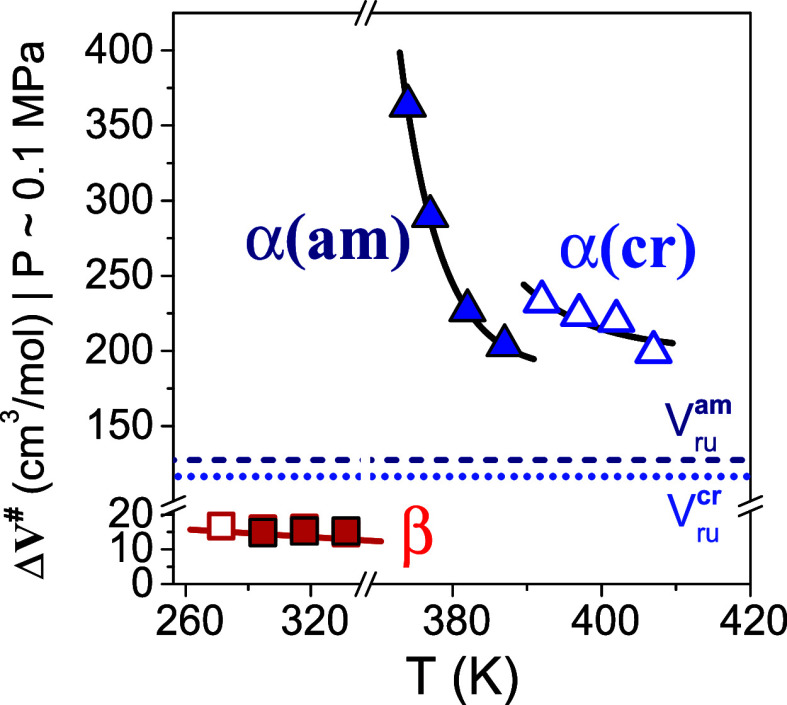
Apparent activation volume (ΔV^#^) as a function of temperature for the α-process in the quenched amorphous state (filled blue triangles), α-process in the semicrystalline state (open blue triangles), β-process in the amorphous state (filled red squares), and β-process in the semicrystalline state (open red squares). The dashed and dotted lines denote, respectively, the repeat unit volume, *V*_ru_, in the amorphous (ρ = 1.4299 g·cm^–3^),^8^ and in the crystalline state (ρ = 1.565 g·cm^–3^).^38,39^.

Earlier studies revealed that this quantity is directly related to the molecular volume of relaxing species, providing significant information about the molecular scale motions.^31,34−36^ On the other hand, a generalized entropy theory^37^ suggested that Δ*V*^*#*^ is more related to the fragility of glass formation, and under conditions of constant cohesive interaction strength, to the packing frustration (and not to the extent of collective motion). We will return to this point in the discussion of the origin of the β-process. Figure 4 provide with the temperature dependence of Δ*V*^*#*^ for the segmental and local relaxation processes labeled as α-process and β-process in both the amorphous and semicrystalline states.

The high pressure sensitivity of the segmental process in the quenched amorphous sample reflects the higher apparent activation volume, which in addition has the stronger temperature dependence. In the same plot, the volume of the repeat unit in both the quenched amorphous and crystalline states (assuming fully amorphous and crystalline compounds) is depicted with dashed and dotted lines, respectively. As proposed earlier,^31,34−36^ Δ*V*^*#*^ exhibits a strong *T*-dependence in the vicinity of *T*_*g*_ and approaches the corresponding repeat unit volume in the range 70–90 K above *T*_*g*_. Within the semicrystalline state, the apparent activation volume of the segmental process is still high, yet it shows a weaker temperature dependence. This is anticipated by the fact that segments are constrained by the crystalline and RAF domains (see below with respect to the kinetics investigation).

### 3.2 Origin of the β-Process

**Figure 5:**
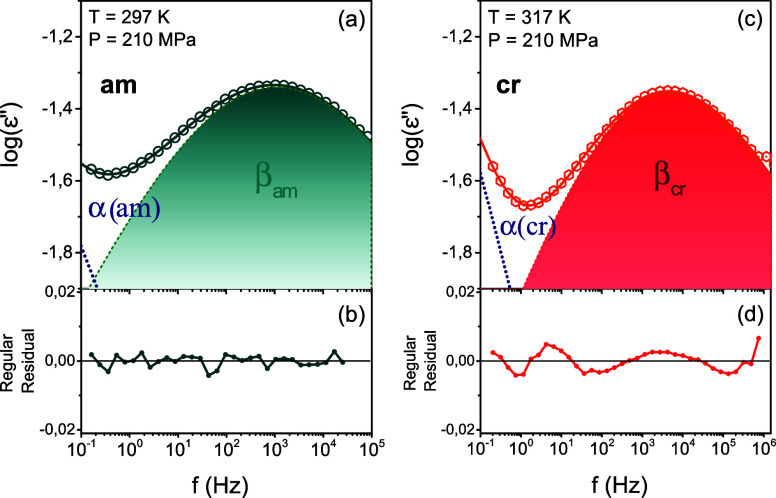
Representative dielectric loss curves of the subglass process in (a) the quenched PEF measured at 297 K/210 MPa, and (c) the semicrystalline PEF obtained at 317 K/210 MPa. The filled areas (quenched: green and semicrystalline: red) in each case correspond to the fitting of the β-process with a single Havriliak–Negami function. Slower processes are the α(am) and α(cr), respectively. (b, d) Corresponding residuals of the fits.

The subglass dynamics in several aromatic polyesters,^16−19,40,41^ display a multimodal character. As an example,^16^ poly(butylene- 2,5 furanoate) (PBF) shows two Cole–Cole processes in the glassy state. The faster was ascribed to the dielectrically active O–C bond of the ester oxygen next to the aliphatic chain, whereas the slower was ascribed to the C–CA link between the ester group carbon and the furan ring. In the case of PEF, the shorter aliphatic segments result to a single–albeit broad–subglass process that can be described by a single Havriliak–Negami process. This is depicted in Figure 5a,b for the melt-quenched PEF at *T* = 293 K and *P* = 210 MPa. On heating to *T* = 313 K, PEF crystallizes, yet the broad subglass process can be described again by a single HN process (Figure 5c,d).

**Figure 6:**
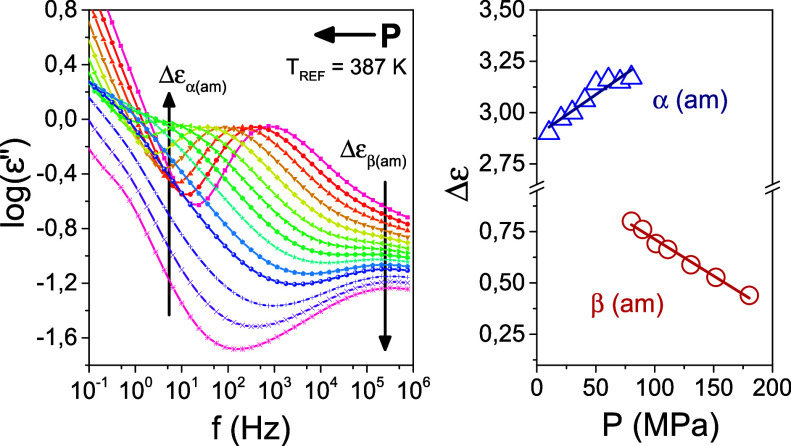
(Left) Dielectric loss curves recorded at 387 K for the quenched melt sample as a function of the pressure. Pressure ranging from 10 to 110 MPa in steps of 10 MPa and from 120 to 180 MPa in steps of 20 MPa. (Right) Dielectric strength as a function of pressure for the segmental mode (blue triangles) and β-process (red circles).

Precise information on the origin of the subglass process in PEF can be obtained by pressure-dependent dielectric spectroscopy measurements. Figure 2c,d, depict the dielectric loss curves of the subglass dynamics for the quenched amorphous and the semicrystalline states, at some selected temperatures. As expected, pressure exerts a stronger effect on the α-process, as opposed to the β-process. In addition, pressurization has a noticeable effect on the dielectric strength of the subglass process in contrast to the minor effect on the shape parameters (Figure S1). This is better shown in Figure 6 for the quenched amorphous PEF at a fixed temperature by increasing the pressure. Increasing pressure has opposite effects on the dielectric strength of the α- and β-processes. This was documented in literature for another class of polymers by Williams and coworkers.^42^ The dielectric strength of the β-process, Δε_β_, is reduced with increasing pressure. This reflects the “blocking” of the corresponding molecular motion, first proposed by Heijboer.^43^ The results are consistent with the notion of an underlying specific molecular motion in the glassy state. In view of this result, it would be interesting to investigate the gas-barrier properties of a pressurized PEF, where the β-process is blocked. Unlike Δε_β_, the relaxation strength of the α-process, Δε_α_^42^ increases with pressure due to the densification of dipoles (Figures 5, S2).

Different from the α-process, the corresponding Δ*V*^*#*^ values for the β-process are very low (, and independent of temperature (Figure 4). The estimated volume of the furan ring flipping is V ≈ 20 cm^3^*/*mol , i.e., directly comparable to the apparent activation volume of the β-process at ambient pressure. It could suggest the flipping of the furan ring as responsible for the β-process of PEF. This molecular assignment of the apparent activation volume contrasts the predictions of the generalized entropy theory.^37^ The blocking of the β-process by pressure suggests some additional experiments, namely, by examining the gas-barrier properties of PEF at elevated pressures. It is anticipated that the suppressed β-process in pressurized PEF would result to even lower CO_2_ permeability.

### 3.3 Phase Diagram

**Figure 7:**
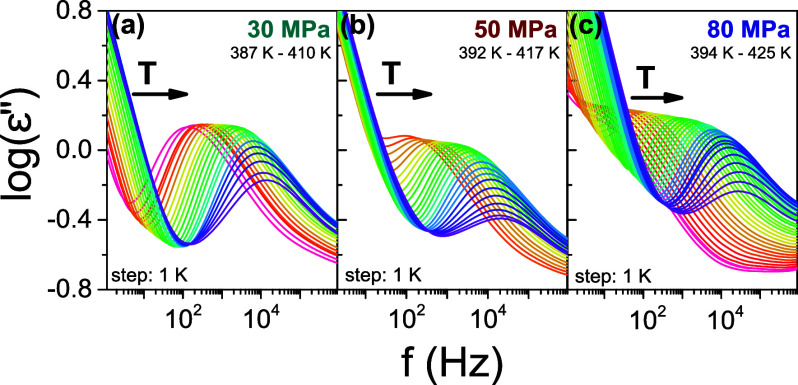
Dielectric loss curves obtained under “isobaric” conditions for the quenched-melt PEF at three different pressures: (a) 30 MPa, (b) 50, and (c) 80 MPa, obtained by slow heating (in 1K steps). Measurements depict the process of cold crystallization by increasing temperature. Cold crystallization is denoted by the reduction in the dielectric strength and concomitant broadening of the α-process.

Here, we employ “isothermal” measurements by increasing pressure (Figures 3, 6) and “isobaric” measurements by increasing temperature (Figure 7) aiming in the construction of the *T*–*P* phase diagram of PEF. The former measurements provide the liquid-to-glass temperature (*T*_g_) by extrapolation to a characteristic relaxation time (τ ∼ 10 s, so as to avoid long extrapolations). The *T*_*g*_ (*P*) exhibits a nonlinear pressure dependence described by the following empirical equation:^44^
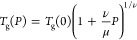
5

**Figure 8:**
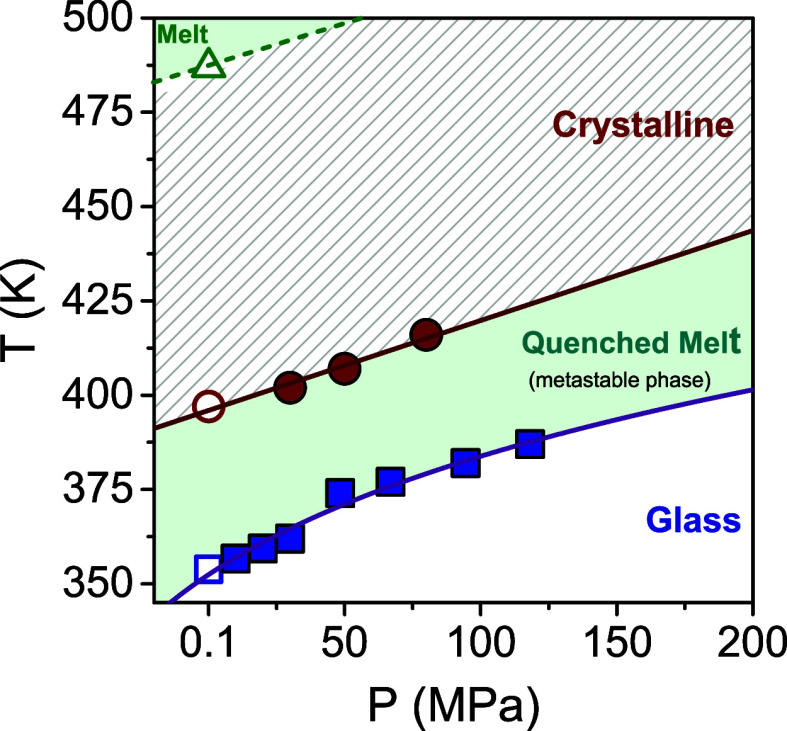
*T*–*P* phase diagram of PEF. It shows four characteristic regimes: “glassy,” “quenched-melt,” “crystalline,” and “normal melt” states. The *T*_g_(*P*) dependence (squares) were obtained from the pressure dependence of the α-process (Figure 3a). The *T*_g_(*P*) dependence (circles) were obtained from the “isobaric” measurements (Figure 7). The line is a result of a fit to the Clausius–Clapeyron equation reflecting the transition from the quenched melt state to the crystalline state. The green dashed line is only an approximation. Open symbols depict the respective temperatures at ambient pressure.

Here, *T*_*g*_ (0) = 353 ± 1 K is the *T*_*g*_ at ambient pressure, and ν and μ are polymer specific parameters (with values ν = 10.4 ± 3.5 and μ = 920 ± 160 MPa^–1^). The pressure coefficient of *T*_*g*_ in the limit of ambient pressure, *dT*_cc_/*dP_|__P_*_→0_, is 383 K·GPa^–1^, a reasonable value for rigid polymers (PS, P2VP, etc.).^31^ “Isobaric” measurements as a function of temperature for the segmental process, were subsequently employed, aiming to identify the transition temperature *T*_cc_(*P*), i.e., from the quenched-melt to the crystalline state. The dielectric loss curves recorded at three different pressures (30, 50, and 80 MPa) by slow heating are shown in Figure 7. Cold crystallization is evidenced by the precipitous decrease in dielectric strength at the *T*_cc_(*P*) (Figures S3, S4). The results of Figure 7, along with the isothermal result, were employed in constructing the pertinent phase diagram (Figure 8). The *T*_*g*_(*P*) and *T*_cc_(*P*) dependencies separate four characteristic regimes corresponding to four different states of PEF, namely, “glassy,” “quenched melt,” “crystalline,” and “normal melt,” by increasing temperature. Concerning the “normal melt” state, a single data point at ambient pressure is attainable due to inaccessible high temperature involved in the pressure investigation.

As can be seen in the phase diagram (Figure 8), the cold crystallization temperature, *T*_cc_(*P*), increases linearly with pressure. Crystallization is a first-order transition, and as such is described by the Clausius–Clapeyron equation as
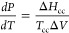
6

Here, Δ*H*_cc_ and Δ*V* are the changes in the enthalpy and volume at the transition, respectively. The pressure sensitivity of the *T*_cc_, is *dT*_cc_/*dP*_|_*_P_*_→0_ ∼ 240 K·GPa^–1^. By employing the Clausius–Clapeyron equation, Δ*H*_cc_ = 45 J·g^–1^ as obtained from DSC (see Figure S6 and S7) and *T*_cc_ = 392 K determined by DS at ambient pressure, the volumetric change at the transition is Δ*V* = *V*_cr_ – *V*_qm_ = 0.028 cm^3^/g

### 3.4 Crystallization Kinetics

**Figure 9:**
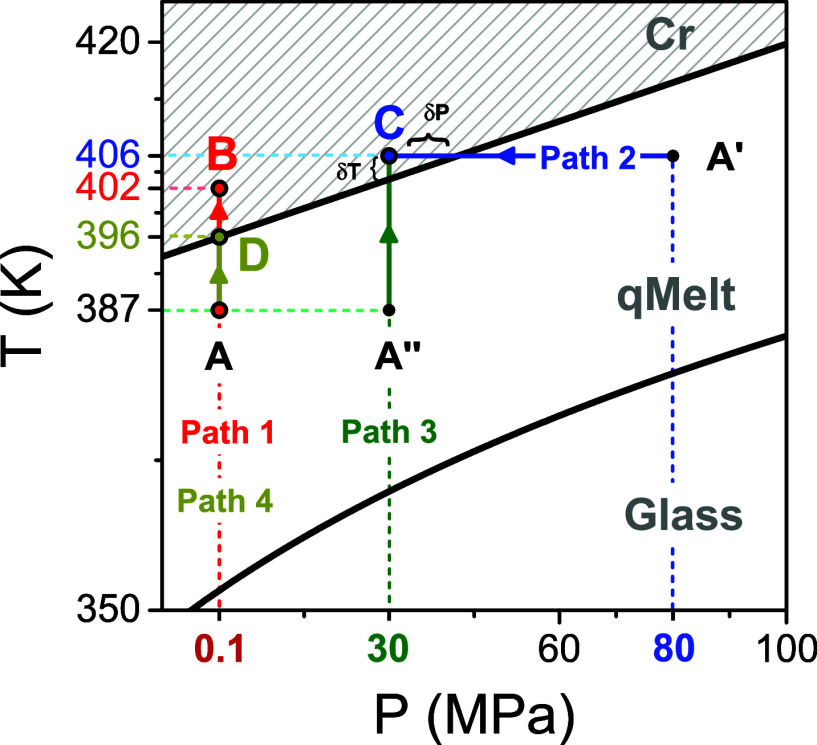
Four different paths within the *P*–*T* phase diagram were employed for the crystallization kinetics of PEF (shown with arrows). In all cases, the sample was initially in the quenched amorphous state. Path 1 (red arrow) describes the temperature jump from A (387 K/0.1 MPa) to B (402 K/0.1 MPa). Path 2 (blue arrow) gives the pressure jump, from A’ (406 K/80 MPa) to C (406 K/30 MPa). Path 3 (green arrow) describes the temperature jump from A″(387 K/30 MPa) to C (406 K/30 MPa). Path 4 (yellow arrow) presents the temperature jump from A (387 K/0.1 MPa) to D (396 K/0.1 MPa).

Investigation of the crystallization kinetics provide the structural/dynamical evolution of crystallizable polymers toward equilibrium, and therefore, is of fundamental interest.^45−49^ Having established the phase diagram,^50^ we followed the crystallization kinetics from the quenched-melt to the crystalline state by different *T*- and *P*-jumps as shown in Figure 9. In particular, **path 1**, from A (387 K/0.1 MPa) to B (402 K/0.1 MPa) was studied via simultaneous SAXS/WAXS measurements, DSC, and DS aiming at a direct comparison of the crystallization kinetics by structural, thermodynamic, and dynamical probes.

**Figure 10:**
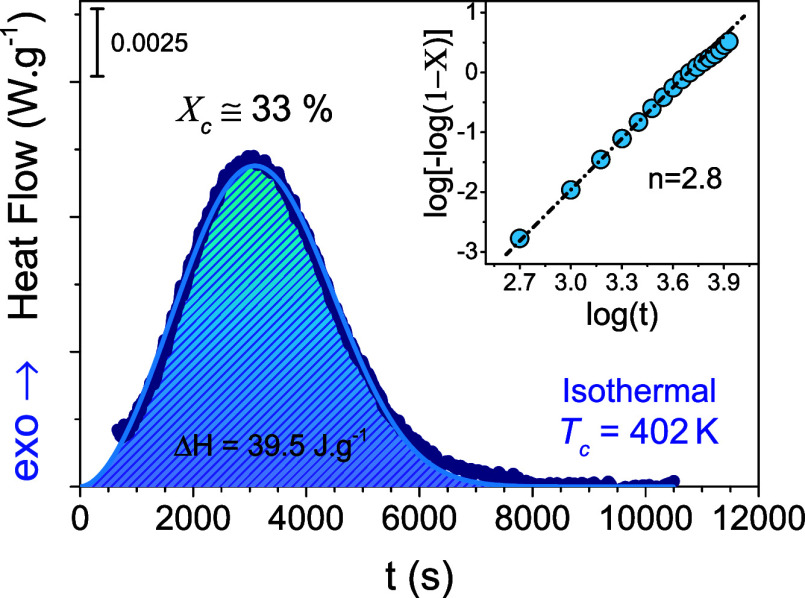
Evolution of heat flow in the isothermally crystallized sample at *T* = 402 K (**path 1**). The light-blue curve is a fit to the experimental data using the derivative of the Avrami function (Weibull distribution). In the inset, the Avrami plot of double logarithmic representation for the crystallization over time is depicted. A linear fit to the initial stages of crystallization provides the Avrami exponent *n* from the slope.

Starting with DSC, we followed the evolution of the heat flow during cold crystallization (Figure 10). Here we monitor the crystallization process from the latent heats at time *t*, Δ*H*_*t*_, in relation to respective heats at *t* = 0 and at the end of the crystallization process, Δ*H*_0_ and Δ*H*_∞_, as
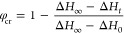
7

The evolution of crystallization follows a sigmoidal curve and can be described by the Avrami equation:^48^
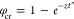
8where *z* is the crystallization rate, *t* is the elapsed time, and *n* is the Avrami exponent. The latter is a function of the type of nucleation process (i.e., thermal or athermal) and the dimensionality of growth. It is more convenient to determine these parameters through the double logarithmic representation as
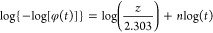
9

By plotting  vs log *t* (inset of Figure 10), *n* and *z* can be obtained, respectively, from the slope and intercept of a linear fit to the data. These values are summarized in Table 1, along with the characteristic crystallization half-times obtained as . We deduce that the Avrami exponent is *n =* 2.8 ± 0.2. It suggests spherulitic growth from athermal nuclei or 2-d growth from thermal nuclei.

**Table 1:** Different–Complementary Methods Used to Obtain the Crystallization Kinetics and the Avrami Parameters during PEF Cold Crystallization from the Quenched-Melt State at 402 K (Path 1)

Method	Parameter	*P* (MPa)	*T*_*c*_ (K)	*n*	*z* (s^–n^)	*t*_*1/2*_ (s)
SAXS	*I*_*max*_(q*)	0.1	402	2.8 ± 0.1	6.0 × 10^–11^± 1.3 × 10^–12^	3900 ± 1100
SAXS	q*	0.1	402	2.8 ± 0.1*	3.8 × 10^–11^ ± 4.3 × 10^–12^	4600 ± 1300
WAXS	*I*_*020*_	0.1	402	2.8 ± 0.1*	6.6 × 10^–11^ ± 5.2 × 10^–12^	3800 ± 1100
DSC	φ_c_	0.1	402	2.8 ± 0.2	1.2 × 10^–10^ ± 3.5 × 10^–12^	3000 ± 1500
DS	Δε (AM)	0.1	402	2.8 ± 0.2	7.8 × 10^–11^ ± 3.1 × 10^–12^	3500 ± 1500
DS	Δε (RAF)	0.1	402	2.8 ± 0.1*	2.1 × 10^–10^ ± 3.4 × 10^–11^	2500 ± 700
DS	φ_c_	0.1	402	2.8 ± 0.15	7.2 × 10^–11^ ± 2.6 × 10^–12^	3700 ± 1600
DS	m (AM)	0.1	402	2.8 ± 0.1*	5.1 × 10^–11^ ± 3.0 × 10^–12^	4100± 1200
DS	*log*(*f*_max_) (AM)	0.1	402	2.8 ± 0.1*	2.2 × 10^–11^ ± 3.2 × 10^–12^	5600 ± 1700
DS	*log*(*f*_max_) (RAF)	0.1	402	2.8 ± 0.1*	3.8 × 10^–11^ ± 2.8 × 10^–12^	4600 ± 1300
DS		0.1	402	2.8 ± 0.1*	4.5 × 10^–11^ ± 6.2 × 10^–13^	4300 ± 1300

*Fixed values.

**Figure 11:**
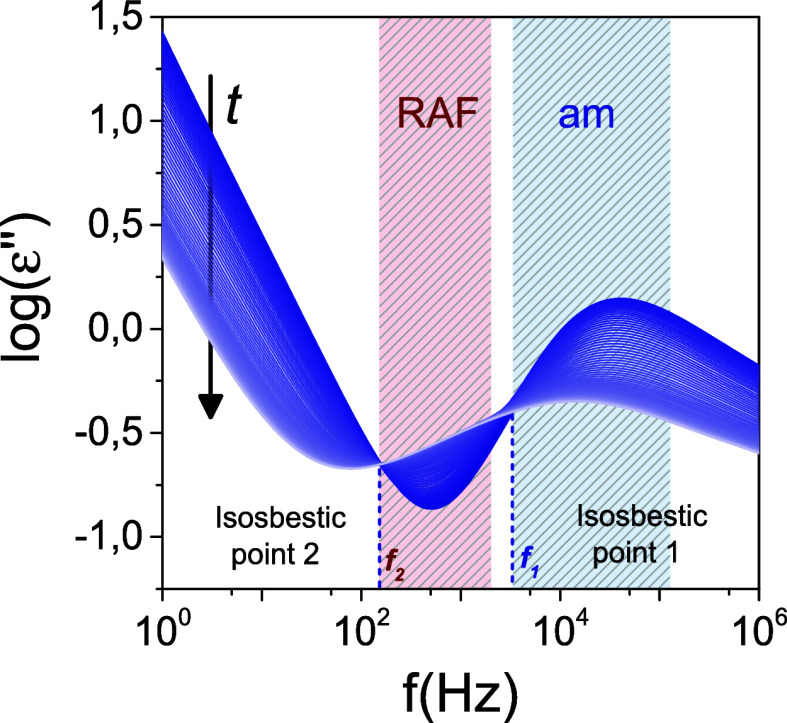
Time evolution of the dielectric loss curves for the quenched sample at crystallization temperature *T*_c_ = 402 K (**path 1**). One can deduce that the dielectric strength of the amorphous process (am) reduces significantly during crystallization, and new processes emerge (the RAF and “slow” processes). The concurrent variations of the three processes (am, RAF, and slow) give rise to two isosbestic points at characteristic frequencies of *f*_*1*_ ≃ 3300 Hz and *f*_*2*_ ≃ 155 Hz.

**Figure 12:**
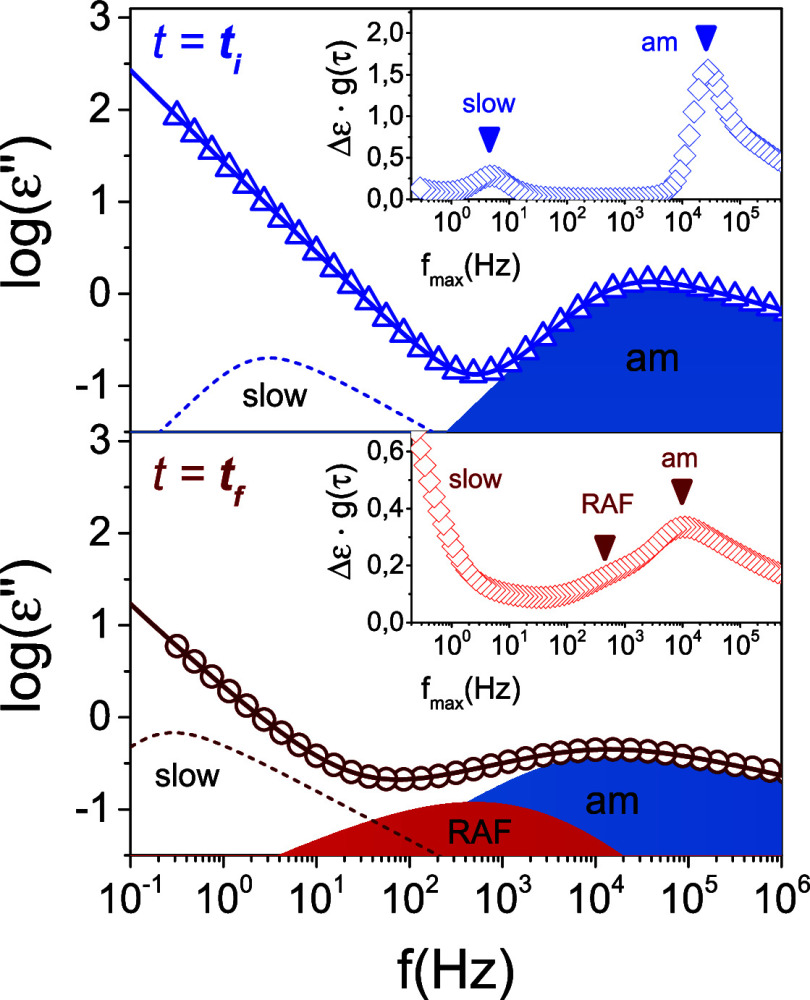
Dielectric loss curves as a function of frequency, for the first *t*_*i*_*=* 104 s (blue triangles) and the last *t*_*f*_ = 10800 s (red circles) measurement (**path 1**), during the isothermal crystallization of the quenched sample at *T*_*c*_ = 402 K. The time interval between two consecutive measurements is *t*_*m*_ = 104 s. At *t = t*_*i*_, a fast process is observed, related to the segmental relaxation of the amorphous sample (am), and a slower one is observed, related to the ionic conductivity. Over time, the sample undergoes cold crystallization, and a new process arises, referred as RAF (restricted amorphous fraction). In the insets, the distribution of relaxation times, Δε·g(τ), is depicted as obtained with GENEREG [52].

The kinetics of cold crystallization were followed dielectrically for the same *T*-jump (path 1) and the evolution of the dielectric loss curves is shown in Figure 11. The figure initially depicts a relatively narrow peak at higher frequencies associated with the segmental (α-) process in the quenched-amorphous state and increasing dielectric losses at lower frequencies due to the ionic conductivity. During crystallization (*T* = 402 K), the peak becomes broader and shifts to lower frequencies. An additional process, associated with the RAF becomes evident (see Figure 12, below) in the course of crystallization. There exist some changes at the lower frequencies, as well. Overall, the kinetics are characterized by two “isosbestic” points as indicated in the figure.,^45−49,51^ This again suggests the presence of several processes with a varying contribution within the experimental frequency window. Because of the slow crystallization kinetics, these processes become distinct only by following the isothermally crystallized sample.

The analysis of the relaxation processes can be made either by employing a summation of HN processes, or equivalently, by employing the distribution of relaxation times from the measured dielectric loss data.^52^ Both methods are employed here, with representative cases shown in Figure 12. The figure depicts the dielectric loss curve all recorded at 402 K (following a temperature jump from 387 K), at *t*_i_ = 104 s, corresponding to the quenched-melt state and at *t*_f_ = 10800 s. At *t*_i_ = 104 s, the curve shows the segmental process in the quenched-amorphous state and a slower process due to the ionic conductivity. Both are evident in the distribution of relaxation times shown as an inset. At the later times (at *t*_f_ = 10800 s) the dielectric loss curves become drastically different. Two relaxations are evident at higher frequencies; one corresponding to the segmental process of segments located in amorphous regions and another to those segments located within the RAF. At the same time, an even slower process appears reflecting the slower dynamics of segments within the crystalline domains (“slow”). This type of relaxation is anticipated on the basis of solid-state NMR measurements in semicrystalline polymers.^53^

In the analysis of the crystallization kinetics, we employed a three-phase model comprising the quenched amorphous fraction (with the corresponding dielectric strength and volume fraction, Δε_αm_ and φ_am_), the RAF (Δε_RAF_ and φ_RAF_), and the crystalline fraction (Δε_cr_ and φ_cr_). The time-dependent dielectric strength of the α-process during crystallization can be described as
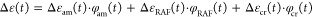
10where, , and extracted the crystalline fraction as
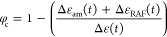
11

**Figure 13:**
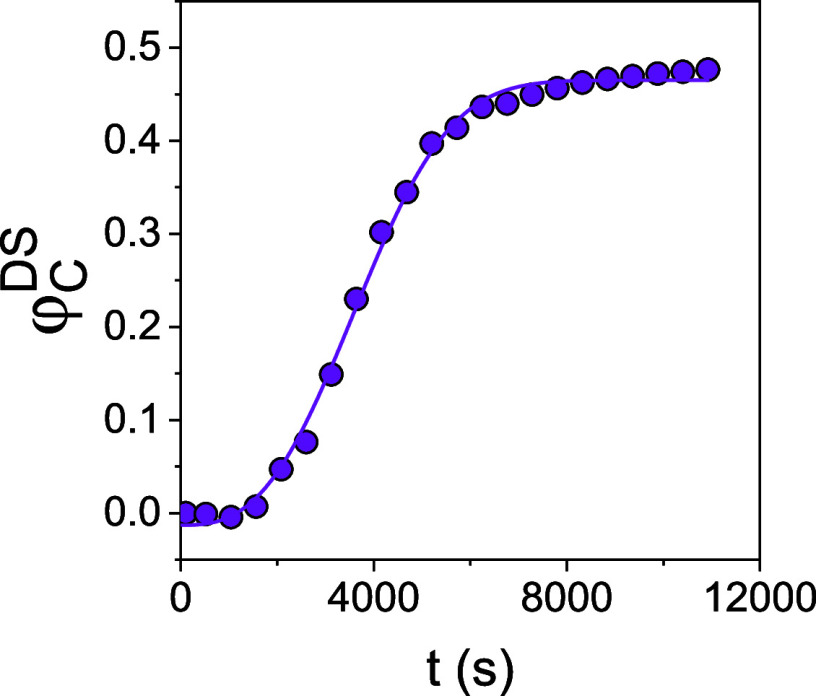
Volume fraction of the crystalline phase calculated from the dielectric strengths of the amorphous and RAF processes, as described in the three-phase model.

The result for the *T*-jump of path 1 is shown in Figure 13. Subsequently, the Avrami equation was employed (eq 8) and the characteristic times are included in Table 1.

**Figure 14:**
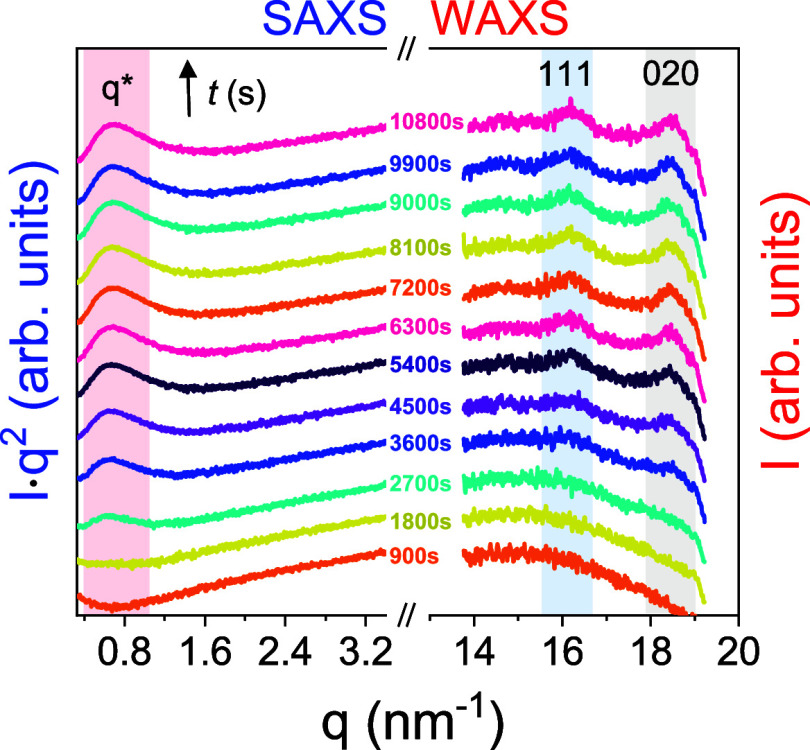
Isothermal SAXS and WAXS patterns of the quenched sample at *T*_*c*_ = 402 K (path1). Curves were shifted vertically for clarity. As the polymer undergoes cold crystallization a peak arises at low *q*, corresponding to the crystalline lamellar at q*. At higher *q* values (WAXS), the cold crystallization of PEF is evident by the emergence of the Bragg reflections (111) and (020) from the triclinic unit cell [39].

Identical experiments were made with simultaneous SAXS/WAXS (**path 1**) as shown in Figure 14. The combined experiment probes two length scales; the formation/evolution of (a) the unit cell (WAXS), and (b) of the crystalline lamellar (e.g., the long period). In SAXS, we obtain the evolution of the SAXS peak at q*, associated with the domain spacing of the crystalline lamellar. In WAXS we obtain the kinetics of the unit cell formation by following the intensity of the 020 reflection associated with the triclinic unit cell of PEF. Ideally, one could obtain the evolution in the degree of crystallinity associated with all XRD peaks. However, this is not possible here because of the small *q*-range available in our setup for the simultaneous measurements.

**Figure 15:**
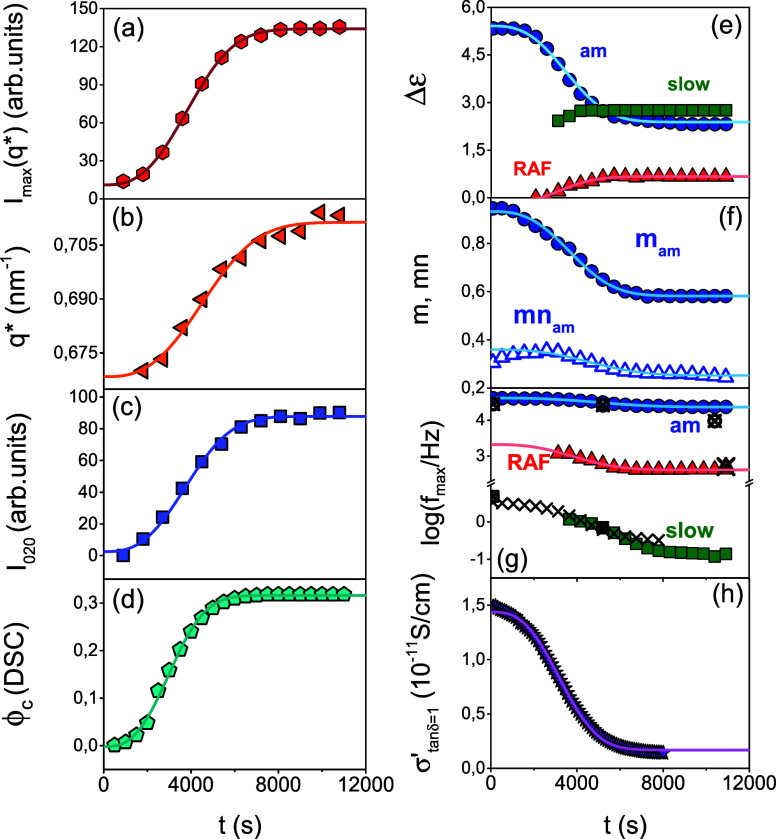
Evolution of several parameters (both “static” and “dynamic”) during PEF cold crystallization from the quenched-melt state at 402 K (path 1); (a) SAXS peak intensity at q*, (b) SAXS domain spacing, q*(*t*), (c) WAXS intensity of 020 reflection, (d) φ_c_ obtained from DSC, (e) dielectric strength of the amorphous (blue spheres), the slow (green tetragons), and RAF (red triangles) processes, (f) low-frequency (*m*; filled spheres) and high-frequency (*mn*; open triangles) HN shape parameters of the amorphous process, (g) characteristic frequency at maximum loss corresponding to the segmental process in the amorphous (blue spheres), the slow (green squares), and RAF (red triangles) processes, and (h) dc-conductivity. In (g), the process due to the ionic conductivity is shown with the × , and the open crossed symbols give the respective frequencies obtained from the distribution of relaxation times, Δε·g(τ) with the software GENEREG. In all cases lines are fits to a corresponding Avrami equation.

The results from the different kinetic experiments are shown in Figure 15. The figure depicts the increase in the intensity of the crystalline lamellar peak *I* (q*), as well as the change in the position of the peak, q*(*t*). The shift in q*(*t*) to higher values during crystallization reflects the decrease in the domain spacing (*d* = 2π/q*) associated with the increased degree of crystallinity. In XRD, the kinetics of the unit cell formation are followed by recording the increase in the intensity of 111 and 020 reflections associated with the triclinic unit cell of PEF. The corresponding Avrami parameters for the structural characteristics are shown in Table 1 and agree within the experimental accuracy. They are also in agreement with the φ_cr_ values obtained by DSC.

Figure 15 in addition to the structural and thermodynamic characteristics contains information about the evolution of dynamics (from DS) during crystallization. The data refer to the evolution of the dielectric strength of the amorphous and RA fractions (Figure 15e). The results suggest a minor RAF at the onset of crystallization that increases with time, a finding that will be discussed below. At the same time, the amorphous fraction (extracted from the dielectric strength of the segmental (α_am_) process) displays a step-like decrease. Both features are in line with the Avrami eq (Table 1). We note here that for the RAF, the Avrami exponent was held fixed to reduce the fitting uncertainty. The figure includes the evolution in the distribution of relaxation times corresponding to the segmental process of the amorphous phase (Figure 15f). It shows considerable broadening of the peak from both the lower (*m*) and higher (*mn*) frequencies. In addition, the peak frequencies corresponding to the relaxation of segments within the amorphous, the RAF and slower (crystalline) processes all shift to lower frequencies (Figure 15g). This suggests that segments within the amorphous peak are dynamically influenced by the increasing crystalline lamellar and RAF growing fractions. Segments in the vicinity of the RAF are more constrained than segments within the center of the amorphous domain. The results could also suggest slow motion of certain segments that are transported from one domain to another, e.g., from the crystalline to the amorphous domains through the RAF, as suggested by solid state NMR in crystal-mobile polymers.^53^ Moreover, we note that at the early stages the slower process is coupled to the ionic conductivity (the latter is shown with the (×)). At later stages, the process seems to have a molecular origin–slow segmental relaxation within the crystalline domains. Lastly, we plot in Figure 15, the evolution of ionic dc-conductivity during crystallization. Ionic conductivity here is very low and extrinsic to the sample (e.g., impurities). Nevertheless, it decreases by an order of magnitude on crystallization. It suggests that ions are exclusively transported via the amorphous phase that is reduced during crystallization.

**Figure 16:**
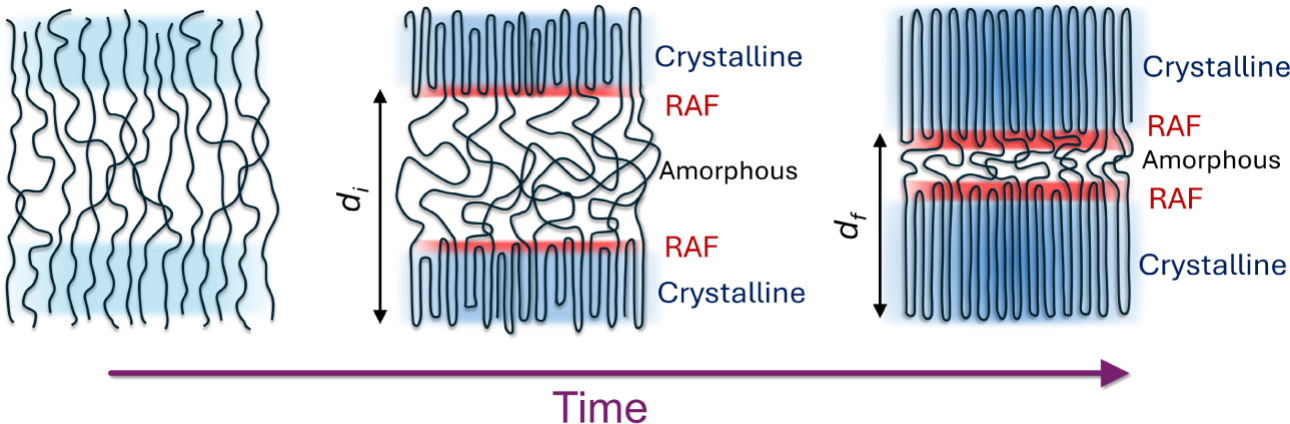
Graphical representation of the structural changes during PEF crystallization. At the initial stages of the crystallization process, only crystalline and amorphous domains are present. The structure resembles that of the mesomorphic phase proposed earlier by Strobl. During isothermal crystallization via path 1, the domain spacing decreases, and a new phase is formed at the boundaries of the amorphous/crystalline domains, the restricted amorphous (RAF). In this view, RAF is coupled to segments participating in chain folding. The RAF increases as crystallization proceeds, following the same kinetics.

Interestingly, the static (SAXS—domain spacing, WAXS—unit cell formation), the thermodynamic (DSC), and dynamic (DS—dielectric strength, distribution of relaxation times, characteristic peak frequencies, and ionic conductivity) probes follow similar kinetics, all of Avrami-type, with exponent *n* = 2.8 ± 0.2 and time scales within the experimental accuracy of the methods. Clearly, they all reflect *different aspects* of the *same* crystallization process. The changes in the amorphous and RAF fractions during crystallization have been employed in the construction of a schematic showing the actual structural changes, in line with the SAXS/WAXS data. The result is shown in Figure 16. It depicts a decreasing amorphous domain, an increasing crystalline lamellar stem, and a concomitant increase of the RAF. The absence of RAF at the early stages of crystallization could imply the existence of an intermediate metastable phase, as suggested by Strobl. In this view, a thin layer with a mesomorphic inner structure is formed between the lateral crystal face and the melt. The first step in the growth process is the attachment of the molten chain sequences to the mesomorphic layer, which subsequently transforms to the crystalline phase. According to the model, the mesomorphic layer comprises locally extended chain segments (in the absence of folding). As time progresses, chain folding appears, creating well-resolved crystal/amorphous domains. This suggests that the RAF is formed exclusively by chain folded segments–where the dominance of gauche conformations and the dual restriction of segments–and grows with time.

**Figure 17:**
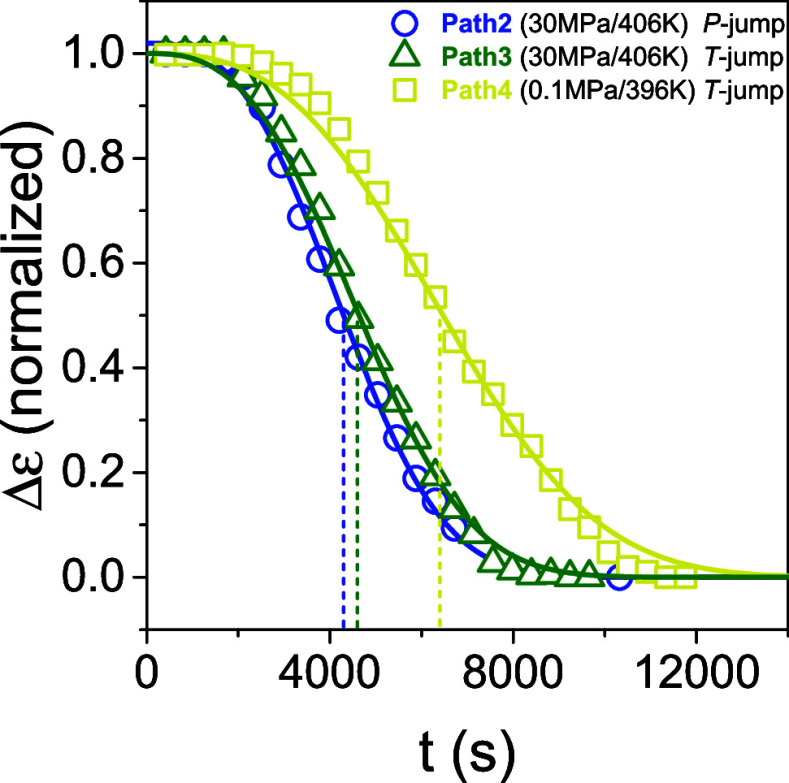
Dielectric strength of the AM process for path 2 (blue circles), path 3 (green triangles), and path 4 (yellow squares), respectively. Path 2 refers to a pressure jump from 80 to 30 MPa, at *T* = 406 K; path 3 describes a temperature jump from 387 to 406 K at 30 MPa; path 4 refers to a temperature jump from 387 to 396 K at ambient pressure. A sigmoidal curve is used to describe the evolution of crystallization, suggesting similar characteristic crystallization times for paths 2 and 3 and a longer crystallization time for path 4 (Table 2).

So far, we explored the PEF crystallization kinetics following path 1. Subsequently, we discuss the crystallization kinetics following different paths in the phase diagram of Figure 9. **Path 2** (blue arrow) represents a pressure jump, from A’ (406 K/80 MPa) to C (406 K/30 MPa). **Path 3** (green arrow) represents the temperature jump from A″ (387 K/30 MPa) to C (406 K/30 MPa). The two jumps have different starting points within the quenched melt state but the same end-point (C within the crystalline state). Lastly, **path 4** (yellow arrow) presents the temperature jump from A (387 K/0.1 MPa) to D (396 K/0.1 MPa). The kinetics of Paths 1 and 4 can be directly compared, as they share the same starting point, but the quench depth is different. Figure 17 compares the evolution of the dielectric strength corresponding to the amorphous process for the three paths. The results of the crystallization kinetics are listed in Table 2.

**Table 2:** Avrami Parameters Derived from the Analysis on the Evolution of the Dielectric Strength for three Different Paths, Path1, Path 2 and Path 4, with Respect to the Phase Diagram (Figure 9)

Probe	Path	Parameter	*P* (MPa)	*T*_*c*_ (K)	*n*	*z* (s^n^)	*t*_*1/2*_ (s)
DS	path 2 (*P*-jump)	Δε (AM)	30	406	2.8 ± 0.1*	4.8 × 10^–11^ ± 1 × 10^–12^	4200 ± 1200
DS	path 3 (*T*-jump)	Δε (AM)	30	406	2.8 ± 0.1*	3.8 × 10^–11^ ± 8 × 10^–13^	4600 ± 1300
DS	path 4 (*T*-jump)	Δε (AM)	0.1	396	2.8 ± 0.1*	1.5 × 10^–11^ ± 3 × 10^–13^	6400 ± 1500

*Fixed values.

According to the crystallization half-time values, *t*_1/2_, (Table 2), paths 2 and 3 are kinetically equivalent suggesting similar quench depths in δ*T* (∼ 3 K) and δ*P* (∼10 MPa). Indeed, state C is located at a hypothetical spinodal line parallel to the Clausius–Clapeyron line (δ*T*/δ*P* ∼ 0.3 K/MPa vs a slope of d*T*/d*P*∼ 0.24 K/MPa). On the other hand, path 4, has the slowest kinetics due to the lowest driving force (the end point D is located in the vicinity of the crystallization line). Especially path 4, with the slow crystallization kinetics suggest ways of keeping PEF in the quenched amorphous state for long time intervals needed e.g., during polymer processing.

## 4 Conclusion

Pressure in addition to temperature-dependent dielectric spectroscopy measurements provided the pertinent *P*–*T* phase diagram of PEF. The diagram comprises four characteristic regimes: “glassy state,” “quenched melt state,” “crystalline state,” and “normal melt.” The glass temperature, *T*_*g*_, had a nonlinear pressure dependence with a strong pressure coefficient, as *dT*_*g*_/*dP*_|_*_P_*_→0_ ∼ 383 K·GPa^–1^, typically found in rigid polymers. On the other hand, the cold crystallization temperature, *T*_*cc*_, increased linearly with pressure following Clausius–Clapeyron, as *dT*_*cc*_/*dP*_|_*_P_*_→0_ ∼ 240 K·GPa^–1^. From the Clausius–Clapeyron equation, we extracted the change in specific volume (Δ*V* = 0.028 cm^3^/*g*) associated with cold crystallization.

Pressure was found to affect the segmental and local dynamics above and below *T*_g_, respectively, in different ways. Increasing pressure increased the dielectric strength of the segmental process (due to densification) but reduced the dielectric strength of the β-process (due to blocking of molecular motion). This finding is consistent with earlier works by Williams and coworkers in another class of polymers.^42^ Furthermore, the low apparent activation volume of the β-process ( is consistent with the flipping of the furan ring as the underlying molecular motion. In general, pressure can affect the gas-barrier properties of PEF through densification (at temperatures above *T*_g_) and blocking of certain molecular motions in the glassy state.

The investigation of the crystallization kinetics from the quenched melt to the cold-crystallized state by a combination of thermodynamic, dynamic, and structural probes provided new insights into the crystallization process. All probes, thermal, structural, and dynamic, followed the same sigmoidal kinetics (with Avrami exponent of 2.8) with comparable time scales. The dielectric study of the isothermally crystallized PEF (at *T*_c_ = 402 K; *P* = 0.1 MPa) revealed three dynamic processes in addition to the ionic conductivity; the segmental relaxation of amorphous segments, the relaxation of segments within the RAF, and a much slower process associated with the constrained relaxation of segments within the crystals. The analyses of the evolution of the dielectric strength for the different dynamic processes revealed the absence of RAF in the early stages of crystallization. This observation is in line with the proposed *mesomorphic phase* by G. Strobl. At later stages, the growth of the RAF followed the same Avrami kinetics as identified by the thermodynamic and structural probes.

Overall, knowledge of the phase state provided experimental routes of keeping PEF in the metastable, quenched amorphous state for long times. This could be of importance in defining new processing conditions of PEF.

## Supporting Information Available

Additional temperature- and pressure-dependent DS data; degree of crystallinity from DSC and XRD for a sample crystallized isothermally at 402 K for 180 min (PDF)
