# Supplementary material for: Phase Diagram, Glassy Dynamics and Crystallization Kinetics of the Biobased Polyester Poly(ethylene 2,5-furanoate) (PEF)
Source: Macromolecules. 2024 Oct 4;57(24):11395–406. doi: 10.1021/acs.macromol.4c01962 (PMC11684343; doi:10.1021/acs.macromol.4c01962)
Supplement: Supplementary file 1 — ma4c01962_si_001.pdf [file ma4c01962_si_001.pdf]

Supporting Information

Phase Diagram, Glassy Dynamics and Crystallization Kinetics of the Bio-based Polyester Poly(ethylene 2,5-furanoate) (PEF)

Ioannis Tzourtzouklis,[1] Panagiotis Kardasis,[1] George Z. Papageorgiou,[2,3] and George Floudas* [1,3,4]

[1]*Department of Physics, University of Ioannina, 45110 Ioannina, Greece*

[2]*Department of Chemistry, University of Ioannina, 45110 Ioannina, Greece*

[3]*University Research Center of Ioannina (URCI)-Institute of Materials Science and Computing, 45110 Ioannina, Greece*

[4] *Max Planck Institute for Polymer Research, Ackermannweg 10, 55128 Mainz, Germany*

Corresponding author: George Floudas (gfloudas@uoi.gr)

ORCID G. Floudas: 0000-0003-4629-3817

Table S1. VFT parameters for the α-process in the amorphous state. Values were obtained from the pressure counterpart of the VFT equation.

T (K)	log(f_{max})	D_P	P_0 (MPa)
370	-1.59 ± 0.06	22.1 *	174 ± 11
373	-1.99 ± 0.05	22.1 *	237 ± 6
378	-2.63 ± 0.03	22.1 *	300 ± 4
383	-3.19 ± 0.02	22.1 ± 4.5	346 ± 3

* fixed values

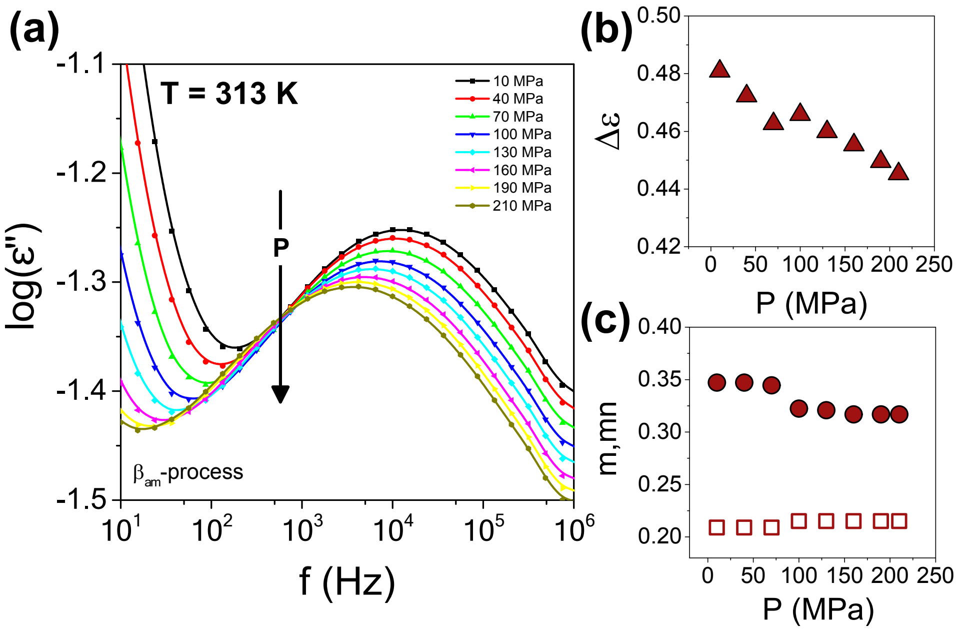

Figure S1. (a) Dielectric loss curves of the β-process in the amorphous state, at the temperature of 313 K and pressure in the range of 10 MPa to 210 MPa, in steps of 30 MPa. (b) Dielectric strength as a function of pressure at the same temperature and pressure range. (c) Shape parameters of the dielectric loss curves, provided by the fit with a HN. With increasing pressure, the dielectric strength is reducing due to blocking of the corresponding motion, while there are no changes in the shape of the curves.

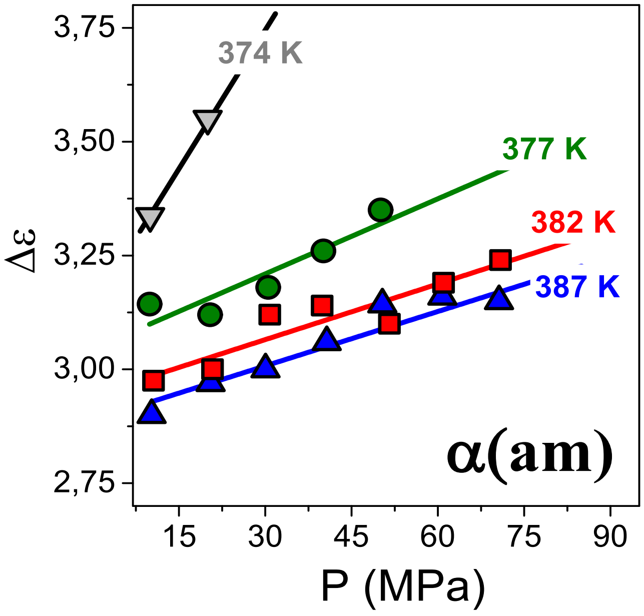

Figure S2. Dielectric strength as a function of pressure for the α-process in the amorphous state. An upward trend is evident due to the increasing number of dipoles per unit volume during pressurization. Different symbols and colors correspond to different temperatures (blue up-triangles: T = 387 K, red squares: T = 382 K, green circles: T = 377 K and gray down-triangles: T = 374 K).

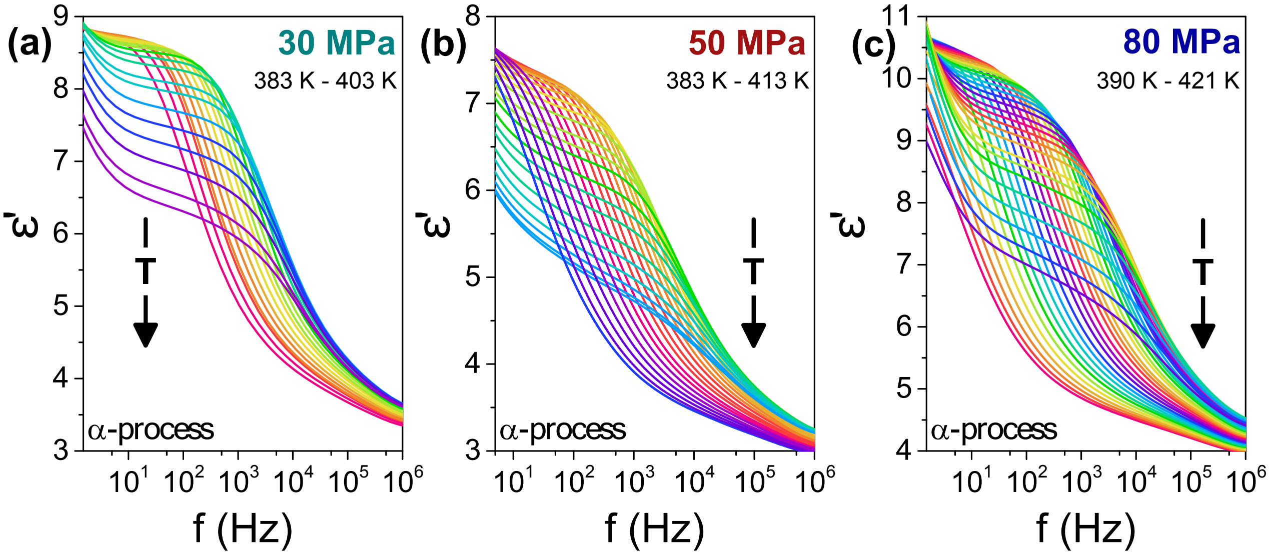

Figure S3. The real part of the dielectric function over frequency for the three different pressures (a) 30 MPa, (b) 50 MPa and (c) 80 MPa. Dielectric strength reduces over temperature as the crystallization process occurs.

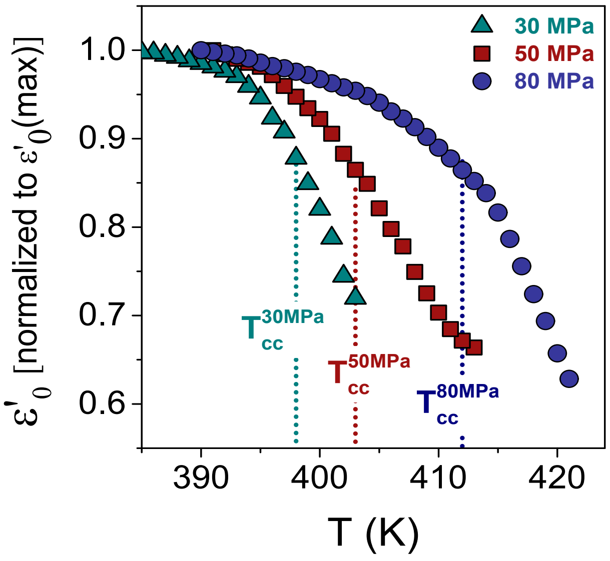

Figure S4. The real part of the dielectric function over frequency for the three different pressures (a) 30 MPa, (b) 50 MPa and (c) 80 MPa. Dielectric strength reduces over temperature as the crystallization process occurs.

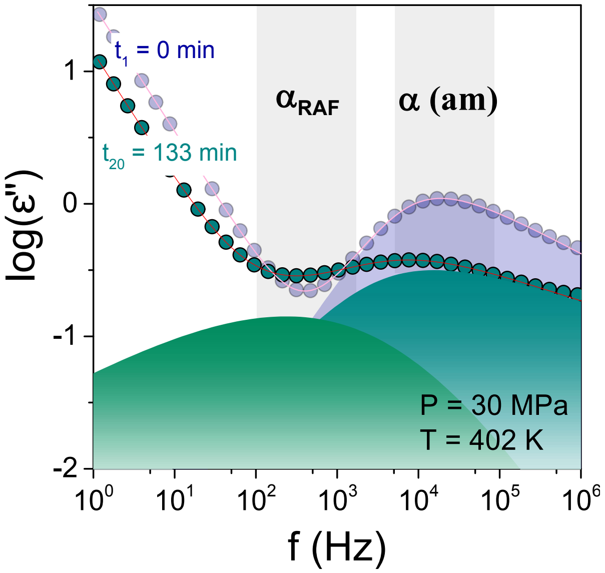

Figure S5. Dielectric loss curves of the α-process at the crystallization point B through path 1. The blurred blue circles correspond to the first loss curve at time t_1=0 min, while the green circles correspond to the loss curve after t_{20} = 133 min. As the time passes the process undergoes cold crystallization and after a long equilibration time a new process arises, denoted as α_{RAF}, originating from the dipoles in the restricted amorphous fraction.

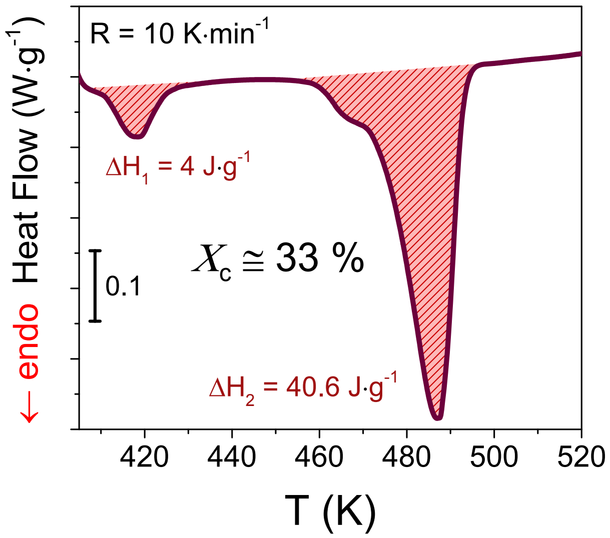

Figure S6. Heat flow over temperature on heating with a rate of 10 K·min^{-1}, following the isothermal crystallization of the quenched sample at T_c = 402 K for t = 180 min. Three endotherms are evident, with a total $\Delta H \simeq 45$ J ·g^{-1}, providing a degree of crystallinity of $X_c \simeq 33\%$ ($X_c = \Delta H / \Delta H_0$, where ΔH_0 = 137 J ·g^{-1} is the heat of fusion for the 100% crystalline PEF) [37].

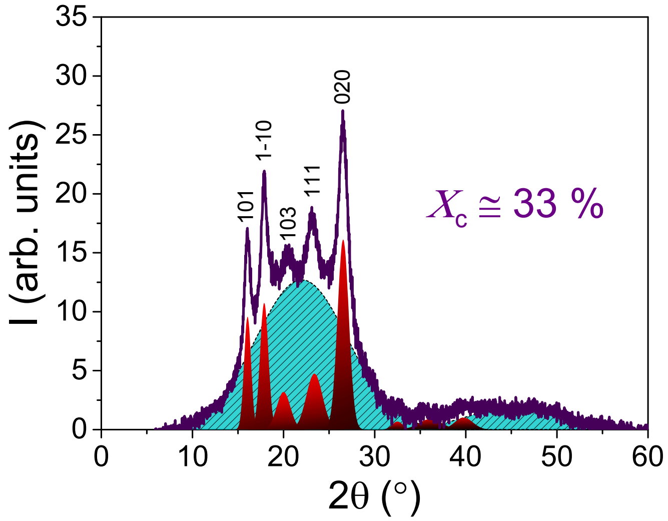

Figure S7. XRD pattern of the crystalline PEF measured at ambient temperature. The sample was previously crystallized over isothermal conditions at T_c = 402 K. Green and red areas correspond to contributions from the amorphous and crystalline peaks. The total degree of crystallinity was calculated to be approximately X_c = 33% in accordance with the value obtained from the DSC.

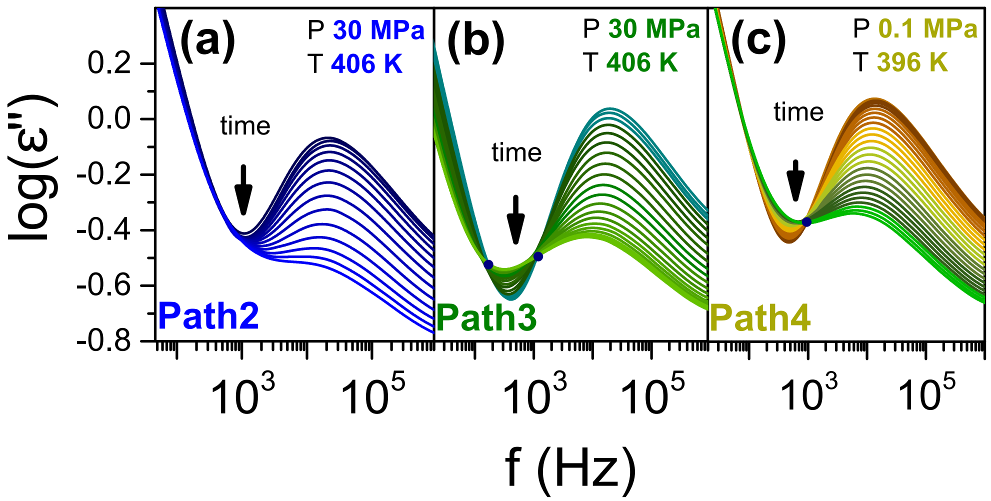

Figure S8. Evolution of the dielectric loss curves for each of the three different paths (a) path 2, (b) path 3 and (c) path 4. The time interval between two consecutive measurements, in all cases, equals 7 minutes. One can observe that crystallization significantly alters the segmental process in both dielectric strength and shape parameters.

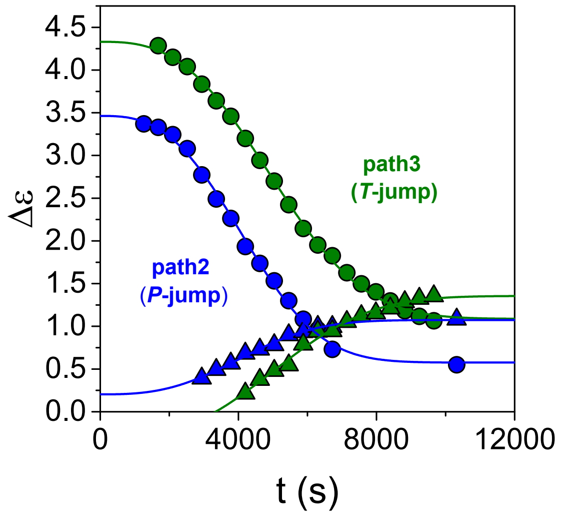

Figure S9. Dielectric strength of the AM (circles) and RAF (triangles) processes for the path 2 (blue) and path 3 (green) respectively. Both paths refer to the same final T and P conditions, describing a pressure jump from 80 MPa to 30 MPa (path 2) at $T = 406$ K and a temperature jump from 387 K to 406 K at 30 MPa (path 3). A sigmoidal curve is used to describe the evolution of crystallization, suggesting similar characteristic crystallization times.
